# Genetic diversity and drug sensitivity profile of *Mycobacterium tuberculosis* among children in Ethiopia

**DOI:** 10.1371/journal.pone.0284363

**Published:** 2023-07-28

**Authors:** Hilina Mollalign, Muluwork Getahun, Getu Diriba, Ayinalem Alemu, Dawit Chala, Begna Tulu, Gobena Ameni

**Affiliations:** 1 National Tuberculosis Reference Laboratory, TB/HIV Directorate, Ethiopian Public Health Institute, Addis Ababa, Ethiopia; 2 Tropical and Infectious Diseases, Aklilu Lemma Institute of Pathobiology, Addis Ababa University, Addis Ababa, Ethiopia; 3 Department of Medical Laboratory Sciences, Bahir Dar University, Bahir Dar, Ethiopia; 4 Department of Veterinary Medicine, College of Agriculture and Veterinary Medicine, United Arab Emirates University, Al Ain, United Arab Emirates; Imperial College London, UNITED KINGDOM

## Abstract

**Background:**

Worldwide, tuberculosis (TB) affects about one million children every year. The burden of the disease is higher in developing countries. However, there is limited information on the lineages and drug sensitivity patterns of *Mycobacterium tuberculosis* (*M*. *tuberculosis*) infecting children in these countries, including Ethiopia. Thus, this study aimed to characterize the different lineages of the *M*. *tuberculosis* complex causing childhood pulmonary tuberculosis and evaluate the drug-sensitivity patterns to the first-line anti-TB drugs.

**Method:**

A total of 54 stored cultures were used in this study. The region of difference 9 (RD9) based polymerase chain reaction (PCR) and spoligotyping were employed for the identification of the isolates at the species and lineages level respectively. Lineage identification was done by using the pre-existing database. Identification of clustering of the spoligotype patterns was by using the SPOLIDB3-based model. The result was retrieved by the most probable family format. Furthermore, the phenotypic, and genotypic drug-sensitivity test (DST) was performed using Mycobacterium Growth Indicator Tube (MGIT™ 960) and GenoTypeMTBDR*plus* assay respectively. Data analysis was done using SPSS version 27 software.

**Result:**

Spoligotyping produced 39 interpretable results for *M*. *tuberculosis*. The majority (74.4%) of them were clustered into 7 groups, while the rest (25.6%) were single. The Euro-American (EA) lineage was the predominant lineage (64.1%) followed by the East-African Indian (EAI) (30.8%) and M. Africanum (5.1%) lineages. The most predominant subtypes were SIT37 (15.4%), SIT149 (12.8%), SIT25 (7.7%), and SIT53 (7.7%). Furthermore, of the identified SITs, T1 and CAS families consisted of 38.5% and 28.2% of the lineages respectively. Drug susceptibility was 91.9% by phenotypic method and 97.4% by molecular assay. The overall prevalence of any resistance was 7.8% and there was a single MDR-TB.

**Conclusion:**

Many of the isolates belong to the modern lineages (Euro American) representing the most common circulating strains in the country. More importantly, despites the tiny isolates tested, drug resistance is low. To fully describe the molecular epidemiology of MTBC lineages in children, we recommend a prospective large-scale study.

## Introduction

Tuberculosis (TB) is among the oldest recorded human infection, which continues to cause illness and death in humans despite the worldwide use of the Bacille Calmette Guérin (BCG) vaccines and different anti-TB drugs [1]. According to the 2020 global TB report by the world health organization(WHO), there were 10 million incident cases of TB, of which 12% occurred among children under the age of 15 [2]. Challenges in bacteriologic confirmation and the poor practice of documentation and underreporting have made the actual estimate of the incidence difficult [3]. These challenges also contribute to the minimal attention for the investigations of TB in children [4].

Exposure to an infectious adult source case with TB were reported as a major risk factor for TB in children [5]. In addition, a higher mortality rate were reported among children with a household contact TB as compared to unexposed children [6]. Therefore, TB in children is a good marker of ongoing transmission in the community [7]. Globally, children experience considerable TB-related morbidity and mortality [8] but, access to the diagnosis and treatment is highly limited in developing countries [9]. Furthermore, clinical diagnostic approaches are nonspecific and microbiologic tests are not sensitive due to the paucibacillary of the samples [10]. As a result, the drug sensitivity profile of TB in children is not well described.

Ethiopia is among the 30 high TB and TB/HIV burden country. In 2019, there was 111,000 notified cases of TB, of which childhood TB contributes 11% [2]. Out of those notified cases, only 65% were bacteriologically confirmed cases. Children in most cases are smear negative and sampling appropriate specimen is challenging. Thus, there is little information regarding the molecular epidemiology of TB in children in this setting. Therefore, this study aimed to characterize the lineages of the *M*. *tuberculosis* complex causing childhood pulmonary tuberculosis in Ethiopia and evaluate the drug-sensitivity patterns to the first-line anti-TB drugs.

## Materials and method

### Study area and source of the isolates

This study was conducted at the Ethiopian Public Health Institute (EPHI), National Tuberculosis Reference Laboratory (NTRL) on stored MTBC isolates. Samples from which the isolates were harvested were collected from selected health facilities in Addis Ababa, Oromia, Amhara and Southern nations and nationalities and peoples (SNNP) of Ethiopia for another study (not published).

### Sampling

The samples were collected by convenient sampling method to enroll all patients presented with sign and symptoms of pulmonary TB (PTB) at the selected study sites till the intended sample size achieved. Fifty-four (54) bacteriologically confirmed cases out of the total 518 presumptive PTB children enrolled across the study sites were treated. This figure does not include smear negative PTB cases treated based on clinical sign and symptom and chest radiography. The current study was conducted on stored clinical isolates of MTBC. The study participants were children 5–15 years of age.

### Study design and study period

A laboratory-based cross-sectional study was conducted using stored MTBC isolates. The study period was from July 2018 to January 2019. A total of 54 media smear-positive cultures were used for this study.

### Inclusion and exclusion criteria

This study has used all cultures that turned positive. All *Mycobacterium tuberculosis* complex(MTBC) isolates presented with a correct patient identification number and having a full demographic data were included in this study. Patients with a negative and contaminated cultures result were excluded from the study.

### Laboratory testing

#### Isolate storage condition

The isolates were stored at -80°C deep freezer. Middle brook 7H9 broth enriched with oleic acid, dextrose, albumin, and catalase (ODAC) was used as a storage media.

#### Purity check

The purity of each isolate was checked by inoculation onto a blood agar plate (BAP)and incubating overnight at 37°C.

#### Subculturing and re-decontamination

Sub-culturing and decontamination were done as previously described in the manual of global laboratory initiatives(GLI) [11]. Pure isolates were sub-cultured by 1:100 dilutions of the original seed before DST setup. Decontamination was performed for positive BAP cultures.

#### Region of difference (RD) 9-based polymerase chain reaction

The identification of *M*. *tuberculosis* from the other members of the *MTBC* species was done using RD9-based PCR as described by Parsons and colleagues [12]. Briefly, RD9-PCR was performed on heat-killed cells to confirm the presence or absence of RD9. Three sets of primers namely, RD9flankF, RD9IntR, and RD9flankR were used. *M*. *tuberculosis* H37Rv, *M*. *Bovis*, and DNA-free water were used as positive and negative controls respectively.

#### Spoligotyping

Spoligotyping was performed as previously described by Kamerbeek and colleagues [13]. The PCR amplification of DNA was done by targeting the direct repeat (DR) locus, the region with the highest level of polymorphism in the *M*.*tuberculosis* genome. The DR region was amplified using primers Dra (GGTTTTGGGTCTGACGAC) and DRb (CCGAGAGGGGACGGAAAC).

#### Lineage classification

The spoligotype patterns were converted into binary and octal formats. Two or more mycobacterial isolates sharing a similar spoligotype pattern were identified as a cluster, whereas single spoligotype patterns were recognized as a singleton. The preexisting database was used to retrieve the spoligotype international type (SIT) species numbers. Spoligotype patterns matching the existing pattern in the SITVIT2 database were identified with the SIT number, whereas spoligotype patterns for which SIT numbers were not found in the database were considered an orphan. Furthermore, the online tool https://tbinsight.cs.rpi.edu/run_tb_lineage.html was used to predict the major lineages and to cluster the spoligotype data.

#### Drug susceptibility test

*Phenotypic drug susceptibility test*. The phenotypic DST was performed using Mycobacterium Growth Indicator Tube (MGIT) against first-line anti TB drugs; Streptomycin (STM), Isoniazid (INH), Rifampicin (RIF), Ethambutol (EMB), and Pyrazinamide (PZA). The DST set consisted of a drug-free growth control tube and one tube for each drug. The critical concentration for each drug was: 1μg/ml, 0.1μg/ml, 1μg/ml, 5μg/ml, and 100 μg/ml for STM, INH RIF, EMB, and PZA respectively. Result interpretation was automatically done by the MGIT machine. This is by comparing growth on the control tube and drug-containing tube. If more than 1% of the test populations were observed in the drug-containing tube, the result was interpreted as resistance to the drug.

#### Genotypic drug susceptibility test (genotype MTBDR*plus* assay)

The chemical DNA extraction method was employed by using the genolyse kit (HainLifescience GmbH, Nehren, Germany). Following the manufacturer’s instructions, the extracted DNA was processed for line probe assay (LPA) using GenoTypeMTBDRplus version 2.0 (Hain Lifescience GmbH, Nehren, Germany). This assay is employed for the detection of resistance-conferring mutations for RIF and INH.

### Quality assurance/control

For every new batch of reagents and culture media, the sterility and performance checks were performed before use. Sterility check was done by inoculation into sterile BAP and performance check was done by inoculation of the control strain (H_37_RV). Positive and negative controls were included in every assay. Data were double entered and cleaned on an excel sheet and exported to SPSS for analysis.

### Statistical analysis

The statistical analysis was performed using SPSS version 27 software. Descriptive statistics were used to depict the demographic variables, spoligotype patterns, and drug-sensitivity patterns. The Pearson’s chi-square and Fisher’s exact test were used to evaluating associations. Associations were considered statistically significant for a p-value of less than 0.05.

### Ethical consideration and consent

Ethical approval was obtained from the Scientific and ethical review board of Aklilu Lemma Institute of Pathobiology with an approval number ALIPB/IRB/011/2017/18. Written informed consent /assent was obtained from parents/guardians and from each study participants. Each study participants were informed as the sample they provided will be used in the future study and the Ethics committee also waived the need of consent for this study. Access to the results and documents were restricted to a secure area to ensure confidentiality. Individual participant information was not disclosed in this study.

## Result

### Demographic and clinical characteristics of study participants

Data describing gender and age is available for 51 and 50 participants respectively. Among these 21 (41.2%) isolates were obtained from female participants. The mean age of the study participants was 10.78 (5–15) years. Most of the positive isolates were obtained from Oromia, accounting for 38.9% (21/54). Most culture-positive study participants (80.4%) were in the age group of 8 to 15 years, as compared to participants in the age group of 5–7 (19.6%) **(Table 1)**.

**Table 1:** Characteristics of study participant from which the isolate was recovered.

Characteristics	Frequency	Percent
Sex		
Male	30	58.8%
Female	21	41.2%
Age		
5–7	7	14%
8–15	43	86%
Residence		
Oromia	24	44.4%
SNNP	13	24.1%
Amhara	9	16.7%
Addis Ababa	8	14.8%
Category		
New	45	83.3%
Retreatment	2	3.7%
Unknown	7	13%

**SNNP**: South Nations Nationalities and peoples

### Mycobacterial isolates

To identify MTBC, 54 stored cultures from PTB patients were sub-cultured using liquid culture. Four isolates were discarded due to contamination, and seven of them were unable to recover after subculture. The remaining 4/54 were non-tuberculous mycobacteria (NTM’s). A total of 39 MTBC isolates were eligible for molecular characterization while, 37 MTBC isolates produced valid result for phenotypic DST.

### Species identification by RD 9- based PCR

**Fig 1:**
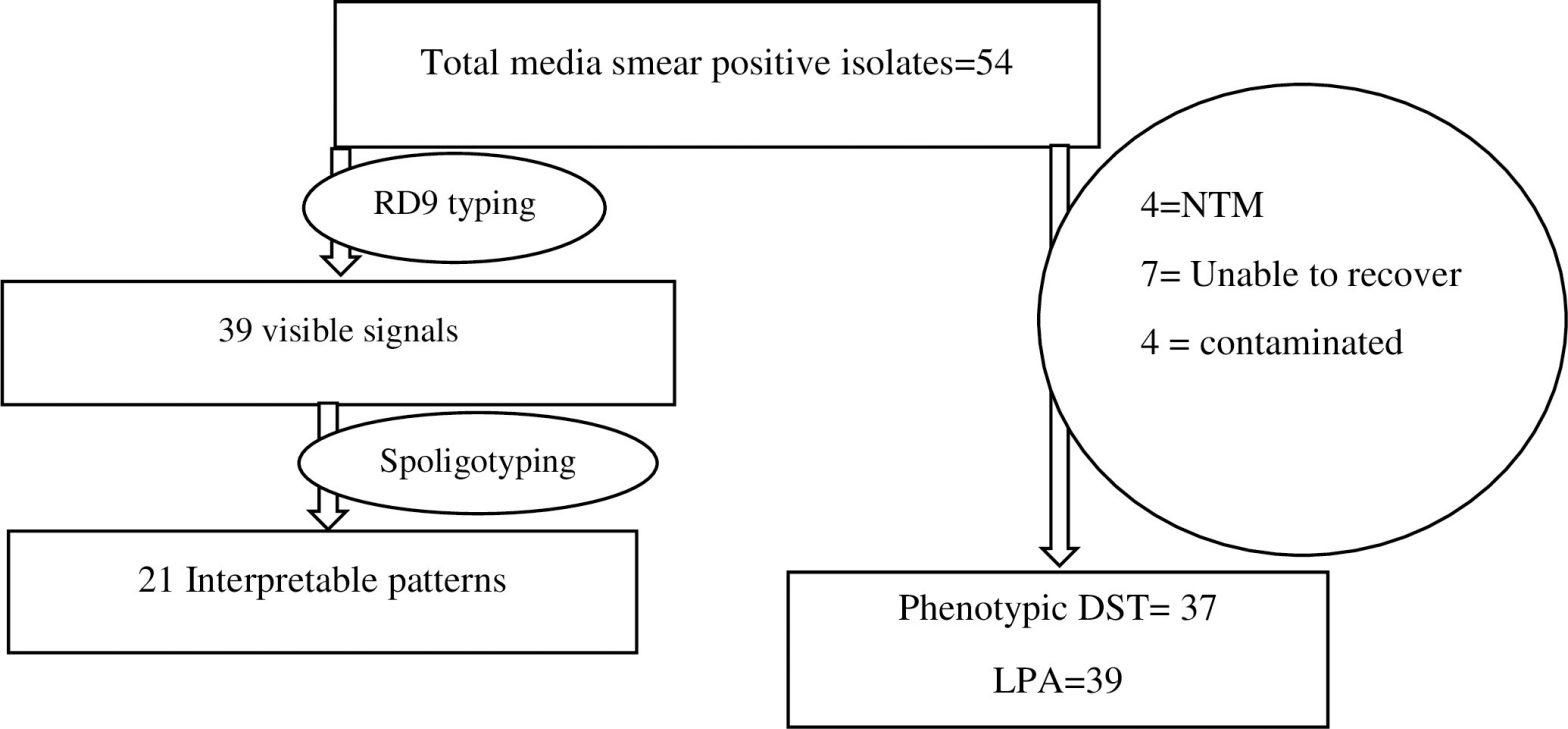
The details of laboratory testing characteristics of the isolates.

All the 54 isolates were subjected to RD 9-PCR for species identification directly from stored cultures before subculturing. The result showed that 39 (72.2%) of the samples were identified as *M*.*tuberculosis*. The remaining had no discernible signal. The details of laboratory testing characteristics of the isolates were demonstrated in **(Fig 1)**.

### Spoligotyping

**Fig 2:**
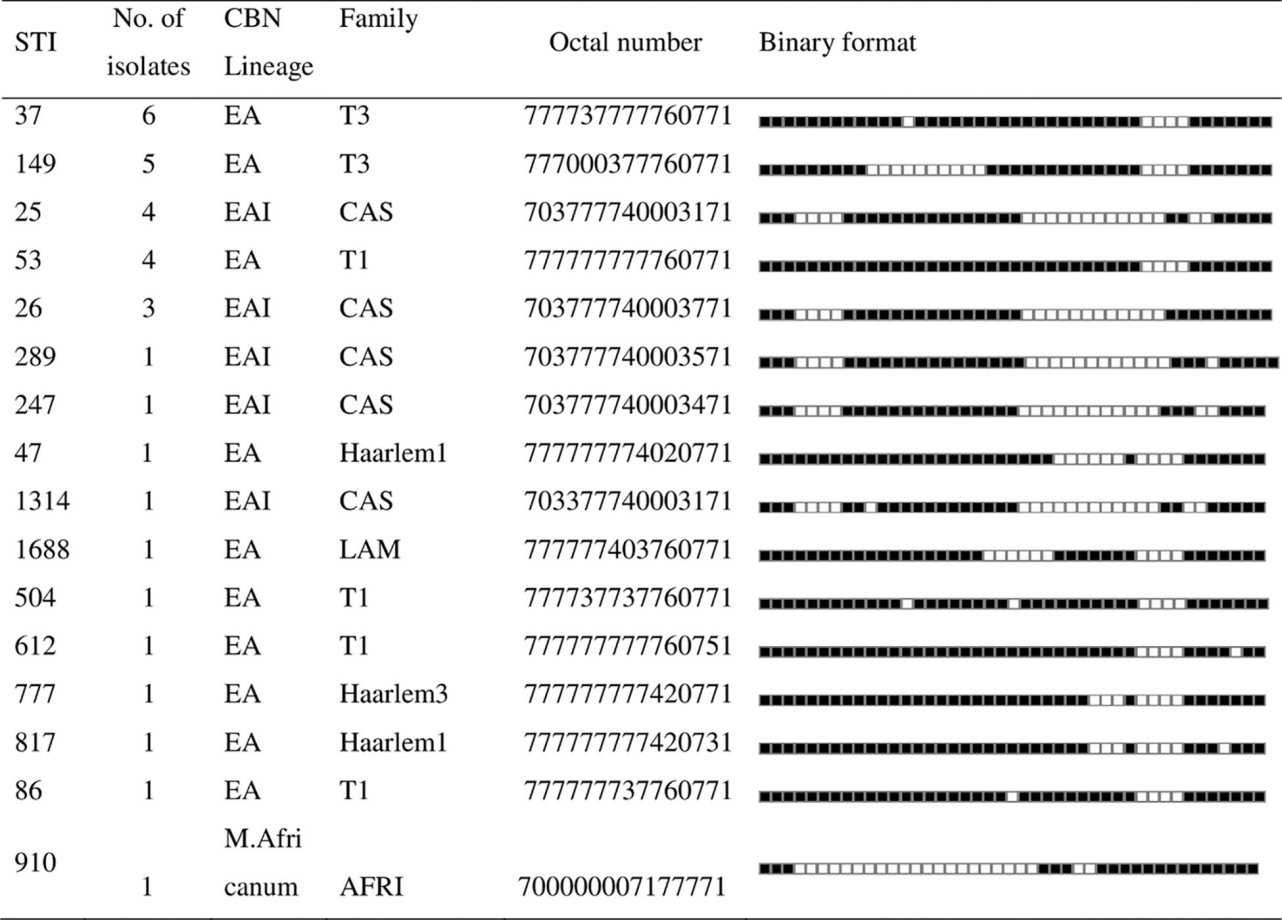
Description of lineages and families of *M*. *tuberculosis* strains isolated from children in Ethiopia (*n*  =  33) for the registered SITs. CAS-Central Asian Strain, CBN: Conformal Bayesian Network; EA-Euro American, EAI-East African Indian, SIT-Shared International Type.

Spoligotyping of 39 isolates with intact RD9 produced 21 interpretable patterns. Of these 21 patterns, 74.4% were clustered into 7 groups, while the rest 25.6% appeared unique. The overall diversity of the isolates was 53.8%. Of the 21 different spoligotype patterns, 15 were assigned to different SIT, whereas 6 patterns were orphans. The dominantly identified SIT were SIT37(15.4%), SIT149(12.8%), SIT25(7.7%), and SIT53(7.7%) **(Fig 2)**.

**Fig 3:**
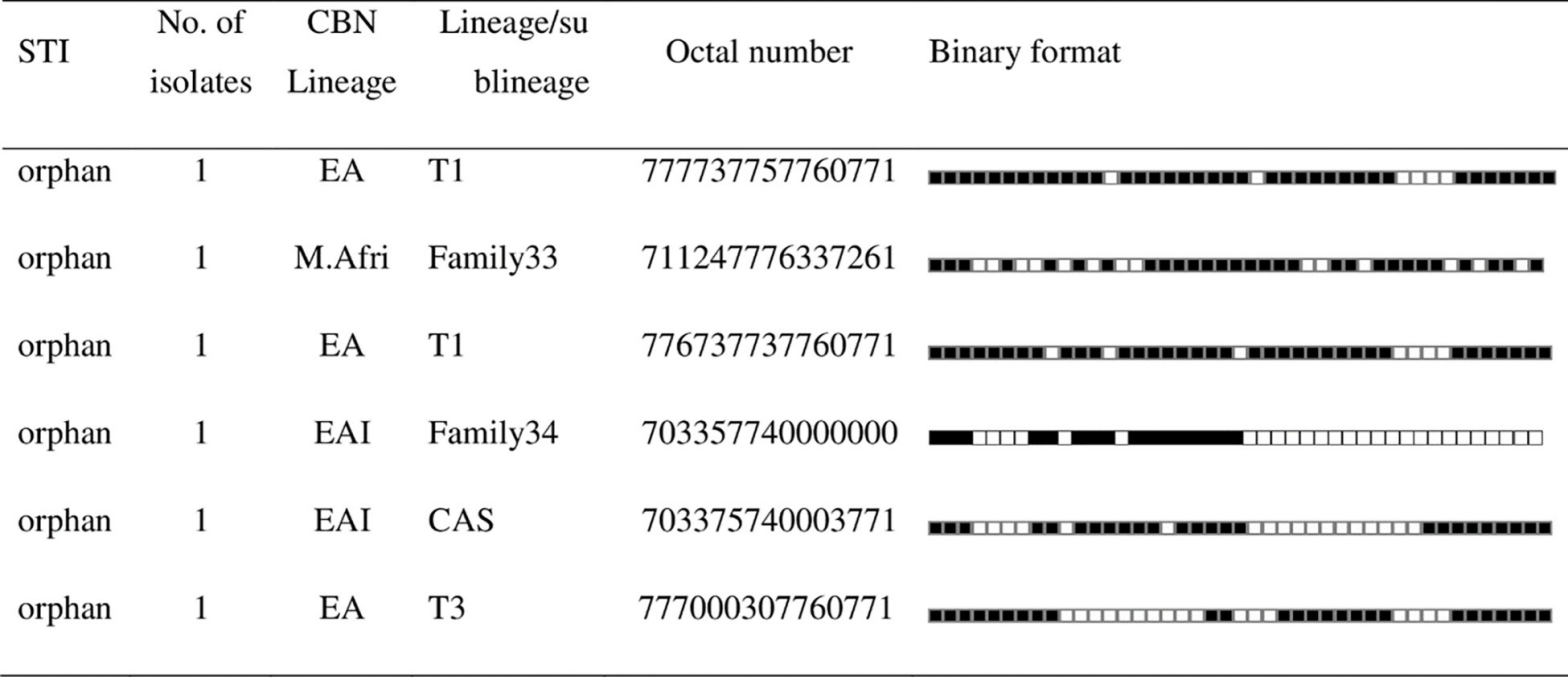
Description of orphan strains among *M*. *tuberculosis* strains isolated from children in Ethiopia (*n*  =  6). CAS-Central Asian Strain, EA-Euro American, EAI-East African Indian, SIT-Spoligotype International Type.

The classification of the spoligotype patterns using TB-insight RUN TB Lineage revealed that the Euro-American(EA) lineage (64.1%) was the most prevalent, followed by the East-African-Indian(EAI) (30.8%) and M.Africanum (5.1%). Further assigning of the lineages into families using the SPOTCLUST tool revealed that 38.5% belong to the T1 family, 28.2% to the CAS family, and 15.1% to the T3 family. In addition, Haarlem1 &3, Family 33,34 &36, as well as LAM9, were also among the identified families **(Figs 2 and 3)**. There were 6 patterns each with a single isolate that did not match the patterns available in the SITVIT database (orphans) **(Fig 3)**.

Of the spoligotyped isolates, 18 (46.2%) were from the Oromia region with a clustering rate of 22.2% (4/18), and 9 (23.1%) were from health facilities in Southern Ethiopia with a clustering rate of 11.1% (1/9). The remaining isolates were from the Addis Ababa and Amhara regions each with 6 patterns and a clustering rate of (1/6) 16.7%. This finding did not show a statistically significant difference in the proportion of clustering across the source sites of the isolates (*p = 0*.*377*). Similarly, there was no significant association between the source site of the isolate with the major lineage identified by CBBN (*p = 0*.*484*). The proportions of occurrence of the dominant lineage (EA) at the sites of South, Oromia, Addis Ababa, and Amhara were 88.9% (8/9), 48% (12/25), 50% (3/6), and 33.3% (2/6), respectively **(Table 2)**.

**Table 2:** Distribution of strains and clustering rate in the study area (*n = 39*).

Characteristics of the isolates	Number (%) of isolates in the study regions				p-value
	Oromia	Amhara	South	Addis Ababa	
Total spolygotyped isolates	18	6	9	6	
					0.377
Clustered	4	1	1	1	
Single	6	3	7	4	
Clustering rate	22.2%	16.7%	11.1%	16.7%	
Major lineage by CBN					0.484
EA	12	2	8	3	
EAI	4	4	1	3	
M.Africanum	2	0	0	0	
The dominant SITs					0.553
SIT 37	4	0	2	0	
SIT 149	3	1	1	0	
SIT 25	0	3	1	0	
SIT 53	1	0	1	2	
Orphans	3	1	1	1	

CBN-Conformal Bayesian Network, EA-Euro American, EAI-East African Indian, IO-Indo Oceanic, SIT-Shared International Type.

### Association of lineage type to drug sensitivity test

We are unable to establish an association between DST profile and lineage type. This is because of the very low mono resistance detected in this study and there is no poly resistance. Further, there was a single MDR-TB and this isolate belongs to the lineage M.Africanum. The majority (56.8%) of sensitive strains for the first-line drugs including PZA and STM belong to the EA lineage while 29.7% to East-African- Indian lineages. Both PZA and STM-resistant isolates are from the EA lineage.

### Drug sensitivity pattern of *M*.*tuberculosis* isolates

Phenotypic(*n = 37*) and molecular(*n = 39*) drug sensitivity test for anti-TB drugs INH, RIF, EMB, STM and PZA (for phenotypic DST) and INH & RIF (for LPA) was performed. Due to contamination and inability to recover from the subculture, the remaining did not undergo DST testing. There were four isolates 7.4% (4/54) of non-tuberculosis mycobacteria (NTM) which were identified by the common mycobacteria/additional species (CM/AS) assay. Two of the NTM species are *M*.*simaie*, while the other two were *M*.*asiaticum*. There was no discordance between the phenotypic and genotypic DST. Mono resistance was observed in 5.4% of the isolates by the phenotypic DST method. This was observed against STR and PZA, each containing a single isolate from newly diagnosed pulmonary TB patients. There was only a single MDR-TB identified by LPA. The MDR-TB was detected in a previously treated case. It was not possible to investigate any association between lineage type and resistance-conferring mutation in the MDR case because of the single number of MDR isolates. Most susceptible isolates were from the new patient category, while only two patients were retreatment cases **(Table 3)**.

**Table 3:** Drug susceptibility profile of the isolates with previous treatment and contact history of the patient.

	New	Retreatment	No contact history	Had contact history
Susceptible	27 (79.9%)	1 (2.9%)	16 (43.2%)	1 (28.2%)
Mono-R	2 (5.4%)	0	2 (5.4%)	0
MDR(LPA)	0	1 (2.6%)		1 (2.6%)

MDR-Multi drug-resistant

Resistance conferring mutation in the MDR isolate was observed on the rpoB and KatG genes. The detected RIF resistance-conferring mutations were the deletion of the WT3 (codons 513 to 517) and WT4 (codons 516 to 519) from the wild type gene and the addition of MUT2B (amino acid change H526D (H445D) or nucleotide change of cac> gac) indicating resistance to Rifampicin. High-level resistance for INH was also observed in which, the WT gene on KatG was deleted and MUT1 (amino acid change S315T or nucleotide change of agc>acc) was inserted.

## Discussion

In the present study, the lineages of *M*.*tuberculosis* infecting children in the study settings were identified and their drug sensitivity testing profiles were described. In this study, we have identified three major lineages through the Conformal Bayesian Network (CBN). These are the EA, EAI, and *M*.*Africanum* lineages. Drug susceptibility testing by the phenotypic method has identified mono resistance for two cases while one MDR-TB in which re-culturing failed to recover live bacteria and was identified by the molecular method. In this MDR isolate, mutation of the WT3 and MUT2B gene conferred resistance for RIF and there was high dose INH resistance. The overall prevalence of any resistance was 7.8% and the prevalence of MDR-TB was 2.6%.

There was a higher clustering rate, while a quarter of the spoligotypes were unique. The most shared types in the cluster were SIT37, SIT 25, and SIT149. This is consistent with previous reports from Ethiopia where these shared types were predominantly reported in the majority of published articles on the molecular epidemiology of TB in Ethiopia [14]. The higher clustering rate and the distributions of clusters of *M*. *tuberculosis* isolates can be an indicator of transmission patterns or the differential fitness of the bacteria [15]. The high degree of clustering and frequency of this shared spoligotype in our study might be an implication epidemiological importance of these strains.

The EA lineage was the dominant lineage, accounting for 64% of the isolates. This finding agrees with the earlier study from Southwest Ethiopia [16]. The 2^nd^ and 3^rd^ lineages under which the isolates were grouped were the EAI and *M*.*Africanum* lineages, respectively. Further characterization of the lineages into families revealed that most of the isolates with SITs reported in this study were dominated by modern clades of the CAS and T families. These families were also reported from central Ethiopia, Afar, and also in the national community-based survey [17–19]. A high proportion of T1 and T3 families were also reported from Debre Berhan, Ethiopia [20]. In addition, a study from Jimma, Ethiopia reported a comparable result from children, suggesting transmission of TB from adults [21]. This result might reflect these specific families might be responsible for tuberculosis infection in children and their epidemiological fitness in different settings. Furthermore, these lineages and sub-lineages might be broadly distributed in the study area from which the source samples were collected. In contrast, a study from Mozambique reported the predominant families of LAM, T, and the globally emerging Beijing clone [22].

According to the drug sensitivity testing results of the isolates, all susceptible isolates were from newly diagnosed TB cases, while only one retreatment case was found to be resistant to INH and RIF. This result is in agreement with the report from Jimma [21], Kenya [23], and a study from the European Union [24]. In contrast, the study from China reported a higher percentage of overall first-line drug resistance in primary cases of TB in children [25]. Our findings suggest that children are becoming infected with drug-sensitive strains, which are easier to treat than drug-resistant strains. Drug-sensitive strains might be transmitted by unidentified contacts. Therefore, the TB control program should strengthen contact tracing through regular training of health care workers.

Mono-resistance for any of the first-line drugs was observed for STR and PZA. Many studies in Ethiopia have reported a high proportion of mono resistance to STR, particularly in adult populations [26]. On the other hand, though PZA is a key drug used to shorten the length of treatment from 9 to 6 months, many studies lack reports of PZA. In our findings, a single case of mono-resistance to PZA was detected in the newly diagnosed TB cases. A study in Vietnam indicates that the proportion of PZA mono-resistance was lower [27]. The global Pooled PZA resistance prevalence estimate was 16.2% [28]. The role of PZA in the diagnosis of drug-susceptible and resistant strains is paramount [29]. Thus, the susceptibility profile of this drug in different populations and settings is important.

This study is not without limitations. The failure of some stored isolates to recover and purify was the main challenge during the lab analysis. The number of isolates tested was also lower which hinders generalizing of the molecular epidemiology of tuberculosis in children. In general, the reported lineages in this study were not different from the previous report from adult populations, which shows the lineages were the predominant circulating lineages in the country. The rate of drug resistance in this study is low. Because of the low number of isolates tested, we cannot generalize to the general child population. However, we can justify that since all MDR cases were managed at the treatment initiating centers (TIC) throughout the country, the probability of a child having contact with MDR adult cases were minimal. In contrast, drug susceptible adult cases follow their medications under the directly observed therapy(DOT). The probability of household contact to drug susceptible TB index case is higher. Thus, drug resistance in children might be due to acquired drug resistance. This study also did not address children <5 years of age and their HIV status.

## Conclusion

In conclusion, the present study showed predominant lineages infecting children appear like the dominant lineages identified from adult populations in different parts of the country. This genotype represents the most common circulating lineages in Ethiopia. Despite the tiny fraction of isolates tested, drug resistance in children is rare. A larger study is required to provide a comprehensive description of the molecular epidemiology of MTBC genotypes in children.

## Supporting information

S1 File Detailed data of the included studies. (XLSX)

## Abbreviations

BA: Blood agar plate
BCG: Bacille Calmette Guérin
CI: Confidence interval
DRTB: Drug-resistant tuberculosis
DST: Drug susceptibility test
EPHI: Ethiopian public health institute
HIV: Human immunodeficiency virus
INH: Isoniazid
MDR-TB: Multidrug-resistant tuberculosis
MTB: *Mycobacterium tuberculosis*
MGIT: Mycobacteria Growth Indicator Tube
NTM: Nontuberculosis mycobacteria
ODAC: Oleic acid Dextrose Albumin and Catalase
PCR: Polymerase chain reaction
PTB: Pulmonary tuberculosis
PZA: Pyrazinamide
QC: Quality control
RD: Region of difference
RR: Rifampicin resistant
SIT: Spoligotype Shared International Type
STM: Streptomycin
TB: Tuberculosis
WHO: World Health Organization
XDR- TB: Extensively drug-resistant tuberculosis
